# The total dispersal kernel: a review and future directions

**DOI:** 10.1093/aobpla/plz042

**Published:** 2019-09-03

**Authors:** Haldre S Rogers, Noelle G Beckman, Florian Hartig, Jeremy S Johnson, Gesine Pufal, Katriona Shea, Damaris Zurell, James M Bullock, Robert Stephen Cantrell, Bette Loiselle, Liba Pejchar, Onja H Razafindratsima, Manette E Sandor, Eugene W Schupp, W Christopher Strickland, Jenny Zambrano

**Affiliations:** 1 Department of Ecology, Evolution, and Organismal Biology, Iowa State University, Ames, IA, USA; 2 Department of Biology and Ecology Center, Utah State University, Logan, UT, USA; 3 Theoretical Ecology, Faculty of Biology and Preclinical Medicine, University of Regensburg, Regensburg, Germany; 4 School of Forestry, Northern Arizona University, Flagstaff, AZ, USA; 5 Department of Nature Conservation and Landscape Ecology, University of Freiburg, Freiburg, Germany; 6 Department of Biology, The Pennsylvania State University, University Park, PA, USA; 7 Geography Department, Humboldt-University Berlin, Berlin, Germany; 8 Dynamic Macroecology, Department of Landscape Dynamics, Swiss Federal Research Institute WSL, Birmensdorf, Switzerland; 9 Centre for Ecology and Hydrology, Benson Lane, Wallingford, Oxfordshire, UK; 10 Department of Mathematics, University of Miami, Coral Gables, FL, USA; 11 Department of Wildlife Ecology and Conservation & Center for Latin American Studies, University of Florida, Gainesville, FL, USA; 12 Department of Fish, Wildlife and Conservation Biology, Colorado State University, Fort Collins, CO, USA; 13 Department of Biology, College of Charleston, Charleston, SC, USA; 14 School of Earth Sciences and Environmental Sustainability, Northern Arizona University, Flagstaff, AZ, USA; 15 Department of Wildland Resources and Ecology Center, Utah State University, Logan, UT, USA; 16 Department of Mathematics and Department of Ecology & Evolutionary Biology, University of Tennessee, Knoxville, TN, USA; 17 Department of Biology, University of Maryland, College Park, MD, USA; 18 School of Biological Sciences, Washington State University, Pullman WA, USA

**Keywords:** Defaunation, dispersal vector, frugivore, mathematical modeling, seed dispersal, seed dispersal effectiveness, total dispersal kernel, total effective dispersal kernel, wind

## Abstract

The distribution and abundance of plants across the world depends in part on their ability to move, which is commonly characterized by a dispersal kernel. For seeds, the total dispersal kernel (TDK) describes the combined influence of all primary, secondary and higher-order dispersal vectors on the overall dispersal kernel for a plant individual, population, species or community. Understanding the role of each vector within the TDK, and their combined influence on the TDK, is critically important for being able to predict plant responses to a changing biotic or abiotic environment. In addition, fully characterizing the TDK by including all vectors may affect predictions of population spread. Here, we review existing research on the TDK and discuss advances in empirical, conceptual modelling and statistical approaches that will facilitate broader application. The concept is simple, but few examples of well-characterized TDKs exist. We find that significant empirical challenges exist, as many studies do not account for all dispersal vectors (e.g. gravity, higher-order dispersal vectors), inadequately measure or estimate long-distance dispersal resulting from multiple vectors and/or neglect spatial heterogeneity and context dependence. Existing mathematical and conceptual modelling approaches and statistical methods allow fitting individual dispersal kernels and combining them to form a TDK; these will perform best if robust prior information is available. We recommend a modelling cycle to parameterize TDKs, where empirical data inform models, which in turn inform additional data collection. Finally, we recommend that the TDK concept be extended to account for not only where seeds land, but also how that location affects the likelihood of establishing and producing a reproductive adult, i.e. the total effective dispersal kernel.

## Teaser

The total dispersal kernel (TDK) describes the combined influence of all primary, secondary and higher-order dispersal vectors on the overall dispersal kernel for a plant individual, population, species or community. Understanding the role of each vector and their influence on the TDK is critically important for being able to predict plant responses to a changing biotic or abiotic environment, yet few examples of well-characterized TDKs exist. Here, we review existing challenges and recent advances in empirical, modeling and statistical approaches to studying the TDK, and suggest ways forward.

## Introduction

**Figure 1.**
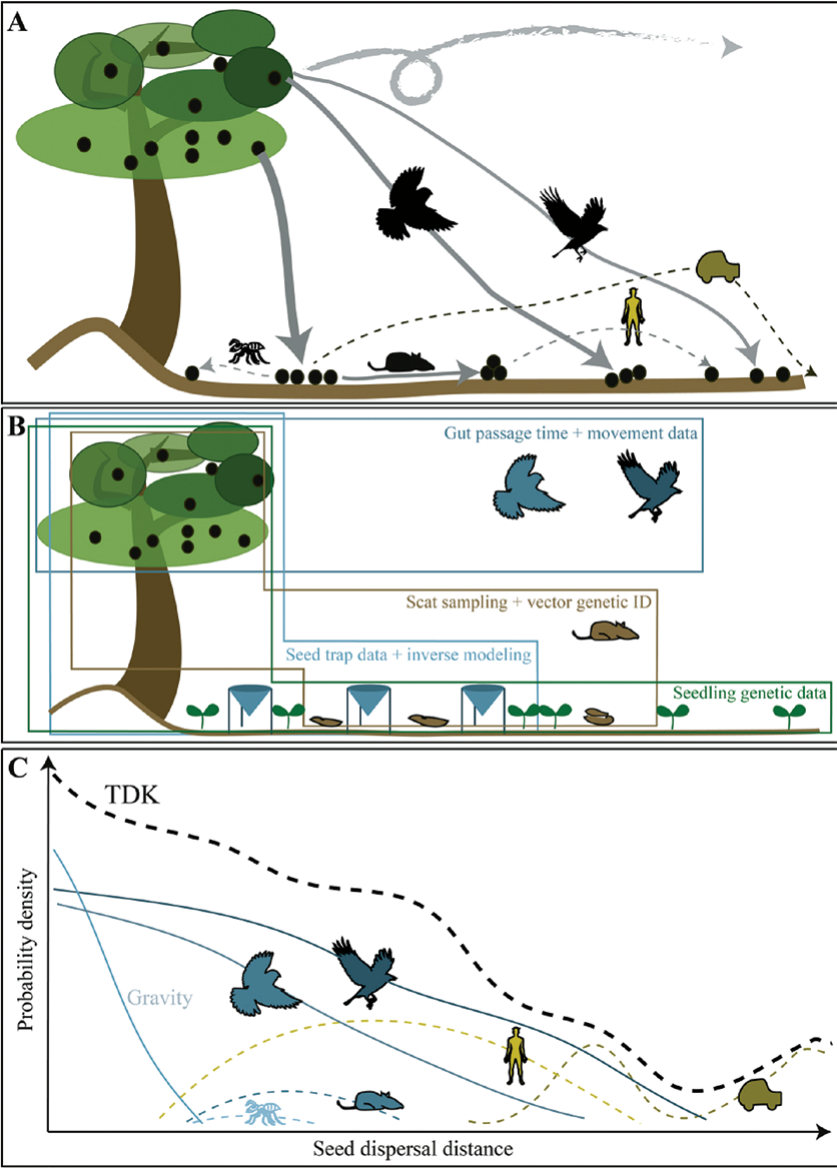
Seed dispersal of one plant species by different vectors (A), methods to assess seed dispersal (B) and the TDK resulting from seed dispersal kernels of different vectors (C). (A) Seeds from a plant can be dispersed naturally by different biotic and abiotic vectors, resulting in varying densities of seeds transported over varying distances. This can include higher-order dispersal, where already dispersed seeds are moved by a subsequent dispersal vector (e.g. scatter-hoarding rodents). Human-mediated dispersal (e.g. by hikers or vehicles) can contribute to seed dispersal, but dispersal distances become more unpredictable since the vector’s travel distances cannot be inferred from its biology alone. The size of icons and thickness of arrows correlate with the number of dispersed seeds—the larger the icon, the more seeds the vector disperses. Different lengths of arrows symbolize varying dispersal distances. (B) Seed trap data combined with inverse modelling incorporates all aerial vectors but does not allow identification of individual vectors and often ignores secondary seed dispersal. Scat sampling with genetic identification allows identification of individual vectors but ignores gravity and secondary dispersal. Both methods may underestimate long-distance dispersal unless genetic approaches are used to match seeds to adult plants across larger areas. Gut-passage time combined with movement data, or tracking of individual seeds from a source plant, can characterize where mobile vectors or wind move seeds but ignore other vectors (e.g. gravity, ants). These three broad approaches need to be combined with seed fate-focused studies to understand seed dispersal effectiveness. Methods that combine genetic data of established seedlings to adult plants can be used to characterize the total effective dispersal kernel (at least to the seedling stage), but do not allow vector identification. Ultimately, a combination of methods will lead to the best representation of the TDK, but also bring challenges associated with integrating different types of data. (C) Conceptualized TDK, including the seed dispersal kernels of all potential dispersal vectors for a given plant species/population. The contribution of each dispersal vector to the TDK depends on its importance, i.e. how many seeds it disperses and how far. Solid lines represent dispersal kernels of vectors where there is empirical evidence for the kernel shape and their contribution to the TDK can be calculated. Dashed lines represent dispersal kernels that have so far rarely been described or studied and their contribution to the TDK is unknown (for example secondary dispersal or human-mediated dispersal), increasing the uncertainty of TDK calculations and illustrating the need for more empirical studies on neglected dispersal vectors. The black dashed line represents the TDK based on all dispersal vector contributions, where contributions of primary vectors are summed and multiplied for secondary vectors. The probabilities densities are scaled such that the area under the TDK is one.

Dispersal is a central demographic process with implications for population persistence, spatial spread, gene flow and community dynamics (Nathan and Muller-Landau 2000; Levin *et al*. 2003; Levine and Murrell 2003). For plants, dispersal is typically characterized using a dispersal kernel, or a probability density function describing where diaspores (referred to as ‘seeds’ henceforth) land relative to the source. This is typically depicted with a two-dimensional probability density function representing the distance from a source and assuming equal probability of traveling in all directions (Clark *et al*. 1998), but could include directional dispersal (van Putten *et al*. 2012), a third dimension (e.g. height, for epiphytic plants) or interactions with properties of the landscape (Neupane and Powell 2015). Different seed dispersal kernels can arise for the same plant species, depending on the vectors involved (Nathan 2007). Seeds can be dispersed by many different vectors and can be re-dispersed several times until they are deposited in their final location (Fig. 1a). While this complexity has long been recognized in the natural history literature, most ecologists still measure dispersal kernels associated with individual dispersal vectors of a particular plant, or measure dispersal for all vectors of a certain type (e.g. volant dispersers) but fail to parse out the role of each vector.

To describe the combined dispersal kernel originating from the mix of the different dispersal vectors, Muller-Landau *et al*. (2003) coined the phrase ‘total dispersal kernel’ (TDK), which was then popularized by Nathan (2007). If there is only a single dispersal vector, then the TDK is equivalent to the dispersal kernel associated with that vector; however, we expect that closer examination will generally reveal multiple movement pathways. Although the TDK concept can be applied to organisms in any taxonomic group, we focus on dispersal of the seeds of plants, the system in which this concept was formally developed (Nathan 2007). TDKs can be used to describe dispersal of individual plants, plant populations, species or communities. Individual plant dispersal kernels scale up to produce the population-level TDK, and population-level TDKs combine to produce a species-level TDK. The most comprehensive TDK is that of an entire community, or a single dispersal kernel that describes the pattern, including variation, of seed rain for all plant species and their dispersal vectors within the community, which may be useful for comparing dispersal patterns across communities or predicting the community-wide impact of defaunation. The TDK both describes the movement of seeds over the landscape and emerges from the properties of the local landscape at a given point in time, so caution is required when scaling up, as there is not a single fixed TDK for an individual, plant, population, species or community.

Total dispersal kernels may be extended to incorporate successful establishment in the form of ‘total effective dispersal kernels’ (TEDK; Schupp *et al*. 2010), or a dispersal kernel combined with a probability density of seedling establishment with respect to distance and direction. This requires additional effort to monitor the influence of vectors throughout the dispersal process (e.g. treatment of seeds, directed dispersal) and subsequent establishment (e.g. identity of neighbours, degree of clumping).

The importance of understanding the TDK extends far beyond the immediate field of dispersal ecology. An understanding of the vectors that contribute to the TDK is needed for modelling the sensitivity of plant species or communities to changes in vectors in response to climate change, over-harvesting, habitat degradation or loss or invasion (Nathan 2007). The concept can be used to compare the diversity of dispersal distances created by all vectors or all plant functional types across different systems, and to examine how sensitive different systems are to changes in spatial or temporal heterogeneity that could arise from landscape or climate change (Mokany *et al*. 2015) or loss or change in biotic or abiotic vectors (Pires *et al*. 2017). TDKs can help identify which plant functional types will be most and least sensitive to climate change by evaluating which vector functional types will be most likely to disperse diaspores long distances with the potential to track changing climate (Bullock *et al*. 2012). Understanding the TDK is also necessary for assessing evolutionary pressures on dispersal (Muller-Landau *et al*. 2003). To be most useful, the TDK should be envisioned as a flexible and generalizable description of seed dispersal with multiple parameters that can be adjusted to reflect the prevailing environmental context.

In what follows, we first summarize the origin of the TDK concept and recent advances. Though the TDK is widely accepted as a concept, we find that it has not been broadly operationalized in the last decade despite its crucial importance for understanding the effects of dispersal on community dynamics. We discuss reasons for this shortcoming and draw on two case studies to demonstrate the value of considering TDKs. We propose possible pathways for overcoming existing empirical, statistical, and modelling challenges and highlight the benefits of addressing these challenges. Overall, we aim to provide a robust and generalizable method for estimating the TDK.

### Origin and recent advances in the TDK concept

Although ecologists recognize the multi-faceted relationships between plant species and their suite of dispersal vectors, they rarely take this complexity into account when calculating dispersal kernels (Nathan 2007). This tendency has persisted over the last decade. A SCOPUS search (26 August 2018) for ‘total dispersal kernel’, ‘complete dispersal kernel’ or ‘full dispersal kernel’ and ‘seed’ in the title, topic or keywords and limited to papers since 2007 returned only two publications (Wichmann *et al*. 2009; Jongejans *et al*. 2015). We included all three search terms because while Nathan uses the term ‘total dispersal kernel’, others have used ‘complete dispersal kernel’ (Dauer *et al*. 2006; Hirsch *et al*. 2012b) and ‘full dispersal kernel’ (Wichmann *et al*. 2009; Hirsch *et al*. 2012b). Wichmann *et al*. (2009) provide an empirical example of a TDK combining primary and secondary dispersal modes. Jongejans *et al*. (2015) develop a gravity model framework that could in principle be used to model the TDK. A Google Scholar search, which scans content in addition to title, abstract and keywords, using the phrase ‘total dispersal kernel’ combined with ‘seed’ and limiting studies to those published since 2007 returned only 17 primary literature papers. Two more papers were returned for ‘complete dispersal kernel’ and eight more for ‘full dispersal kernel’. From these results, it is clear that the phrase (or concept) ‘total dispersal kernel’ has not been widely applied, and that there are multiple terms being used to describe the concept.

That few studies have tried to implement or measure the TDK does not indicate a lack of progress in seed dispersal ecology relevant to the TDK concept. Some noteworthy case studies include Dennis and Westcott (2006), Jordano *et al*. (2007), Pires *et al*. (2017) and Bastida *et al*. (2018). In some systems, researchers have made progress in estimating TDKs without using the term or differentiating between individual vectors. For example, when a plant has a single dispersal vector, then the dispersal kernel is equivalent to the TDK, although the term is not likely to be used. In addition, studies of spatial analysis of trees (e.g. Rue *et al*. 2009), and genetic analyses of seedlings relative to adults (e.g. Hardesty *et al*. 2006) demonstrate the TEDK.

The field has made progress in determining which dispersal vectors contribute to the TDK and the relative importance of their contributions. Data on fruit–frugivore networks have improved, often allowing us to identify the primary dispersal vectors for a community of tree species (de Almeida and Mikich 2018). Previously overlooked dispersal vectors, such as migratory birds, are increasingly incorporated in predicting dispersal kernels (Viana *et al*. 2016). Data collection on migratory birds has mostly been sporadic and opportunistic, but the inclusion of molecular analysis, process-based models, direct observations and distributional patterns of both birds and dispersed plant species can provide more accurate long-distance dispersal (LDD) estimates and hence contribute to the TDK of individual species. The role of seed dispersal by carnivores through predation of seed-dispersing animals, or diploendozoochory, has now been assessed for several plants; their impact on the dispersal kernel and recruitment of these species can be surprisingly relevant (Jordano *et al*. 2007; Hämäläinen *et al*. 2017). We are also gaining a greater understanding of the role of humans as dispersal vectors, which can have a large influence in many systems (Bullock *et al*. 2018). Boxes 1 and 2 provide case studies of systems where the TDK has been explored, with implications for management.

Box 1. **Total dispersal kernel case study 1: *Carduus nutans***

*Carduus nutans* (musk thistle) is a non-native invasive species, appearing on noxious weed lists in many countries for its negative economic impacts (Desrochers *et al*. 1988). Its seeds are putatively dispersed by wind (Desrochers *et al*. 1988), but are also moved by birds, water, vehicles and as a contaminant of agricultural seed (Medd and Smith 1978); thus, significant long-distance dispersal is via human movement. Secondary dispersal occurs via insects and small mammals (Jongejans *et al*. 2015). Furthermore, wind-mediated dispersal of *C. nutans* is affected by: climate warming (Zhang *et al*. 2011; Teller *et al*. 2016); drought (Teller *et al*. 2014); habitat complexity (Marchetto *et al*. 2010); phenotype (Teller and Shea, in preparation); insect attack (Marchetto *et al*. 2014) and environmental conditions at seed release such as turbulence, temperature and humidity (Skarpaas *et al*. 2006; addressed in wind tunnel seed release trials: Jongejans *et al*. 2007; Marchetto *et al*. 2012). Assessing gene flow across the landscape would also require information on pollen movement (Yang *et al*. 2015). Much of these empirical data have been incorporated into statistical dispersal models (Skarpaas *et al*. 2010) and mechanistic models for dispersal and spread of this species (Skarpaas and Shea 2007; Jongejans *et al*. 2008, 2011) with implications for management (Shea *et al*. 2010).

Box 2. **Total dispersal kernel case study 2: *Mariana Islands***

In the Mariana Islands, the majority of forest trees have fleshy fruits adapted for animal dispersal. The island of Guam, however, has experienced the full or functional extinction of all forest bird and bat species by the invasive brown treesnake (*Boiga irregularis*), leading to dispersal failure (Rogers *et al*. 2017). While it would be ideal to restore all frugivores, conservation funds are limited; therefore, it is imperative to recognize the contributions of each frugivore towards the total dispersal kernel. The possible dispersers include five frugivorous birds still present on nearby islands, the Mariana fruit bat, land crabs, as well as non-native frugivorous rats and pigs. We believe ant seed dispersal is negligible, based on the ants present in the islands, however more study is needed for confirmation. To identify the role of each frugivore species, researchers conducted feeding trials and observations of diet in the wild using fecal samples and frugivory observations (Fricke *et al*. 2017, 2019). The movement of frugivores combined with gut passage time informed species-specific seed dispersal kernels (Rehm *et al*. 2019). Dispersal kernels were affected most strongly by the movement patterns of each frugivore rather than the time each frugivore took to pass seeds or the identity of each plant species. In addition, not all frugivores were effective dispersers—the white-throated ground dove (*Alopecoenas xanthonurus*) destroyed nearly all seeds it consumed, as did the black rat (*Rattus rattus*), already a well-known seed predator. The rate of frugivory by each disperser species will need to be combined with the appropriate dispersal kernel to create total dispersal kernels. Some of the challenges in doing this include measuring frugivory rates of nocturnal fruit bats in a manner comparable to that of birds and estimating secondary dispersal rates and distances by pigs. Additional studies have evaluated the quality of dispersal by each disperser, which could be integrated to produce total effective dispersal kernels (Rehm *et al*. 2017). Overall, this research has demonstrated that while the TDK of most forest trees involves multiple vectors, the såli (Micronesian starling, *Aplonis opaca*) is a particularly effective disperser, and a strong candidate for restoring seed dispersal to Guam, especially since a small population has persisted in the northern part of the island. However, rewilding Guam with såli will only be possible if snakes are controlled across significant areas, which may be possible in the near future (Engeman *et al*. 2018).

### Empirical challenges and possible solutions

The fact that few studies have measured a complete TDK is most easily explained by the monumental effort required to measure all aspects of dispersal, including where a seed originated (natal source), the means of dispersal (abiotic and/or biotic vectors), the number of individual dispersal events to which a seed is subject (primary, secondary, tertiary, etc.), its final location and, for TEDKs, the fate of the seed (death or germination, establishment and growth; Fig. 1a).

#### Identifying the seed origin

Common approaches used to fit empirical seed dispersal kernels include Eulerian methods that measure population-level patterns and Lagrangian approaches that consider the movement of individual seeds (Fig. 1b). The first category includes studies that capture seeds around a single source then fit kernels directly (Bullock *et al*. 2017), and those that use inverse modelling to probabilistically link seeds found in seed rain traps to the adult plants found around them (Clark *et al*. 1998; Nathan and Muller-Landau 2000). Additionally, molecular genetic methods and parentage analysis can be used to match seeds or seedlings back to source plants (Ashley 2010), and molecular approaches can aid in identifying the disperser (Jordano *et al*. 2007). The second category includes studies that track individual seeds directly from the source plants (Bullock *et al*. 2006), those that combine animal movement data with gut-passage data (Fig. 1b) to model the probability distribution of seed dispersal distances (Razafindratsima *et al*. 2014; Pires *et al*. 2017) and those that monitor seeds within wind tunnels to document seed release (Skarpaas *et al*. 2006) and inform mechanistic models of wind dispersal. Each of these approaches has advantages and drawbacks when fitting seed dispersal kernels. Data obtained using Eulerian and Lagrangian sampling methods may lead to different TDK estimates, for example, because of temporal autocorrelation in wind speeds during short-term studies (Skarpaas *et al*. 2011). The sampling process should thus be taken into account in TDK analyses.

Inverse modelling and genetic methods used for fitting TDKs typically require knowing the location of all reproductive plants within the area being studied. Simply locating all individuals within a given area can be difficult for moderately common species or for small and cryptic plants, as well as in systems that are physically difficult to navigate or with large areas of continuous habitat. However, a combination of machine learning and remote sensing could generate datasets of plant distributions using automated species identification (Mehdipour Ghazi *et al*. 2017). Species-level plant identification from airborne imaging has been achieved for three focal species in Barro Colorado Island, Panama at 94–100 % accuracy (Baldeck *et al*. 2015). Andrew and Ustin (2010) derived dispersal kernels (in essence, TEDK’s) for an invasive plant using airborne hyperspectral imagery and image analysis. Spaceborne spectroscopy, such as hyperspectral or thermal remote sensing, can also aid in species identification, at least for dominant plant species or functional types across large landscapes (Feret and Asner 2014; Roth *et al*. 2015). To reduce the chance that reproductive adults outside the region of focus contribute seeds via LDD, a pilot study combined with a simulation model can help set appropriate spatial bounds (Bullock *et al*. 2006).

Another challenge is matching seeds caught in seed traps to the maternal source when there are multiple possible seed sources. Inverse modelling approaches do not identify the parent tree, but fit dispersal kernels by assuming each tree has some probability of contributing seeds to each seed trap or seedling quadrat (Clark *et al*. 1998; Nathan and Muller-Landau 2000). Increasingly, parentage analysis is being used instead to trace the origin of dispersed seeds, often with better results (Klein *et al*. 2013). Parentage may be determined through genotyping the endocarp of a seed, which is of maternal origin and therefore matches the genotype of the source tree (Godoy and Jordano 2001). Alternatively, neighbourhood models can be used to identify the paternal pollen source and maternal parent for naturally established seedlings, given genetic data from seedlings and all possible parent plants at the site (Burczyk and Koralewski 2005; Moran and Clark 2011). Genetic analyses have frequently revealed that dispersal distances are much farther than when the nearest neighbour is assumed to be the parent (Jordano *et al*. 2007; Piotti *et al*. 2009; Johnson *et al*. 2017a). Both individual and population assignment methods using genetic approaches (Robledo-Arnuncio and Garcia 2007; Broquet and Petit 2009) have provided valuable insight into LDD, a notoriously difficult process to measure (Cain *et al*. 2000). While microsatellite markers have traditionally been the dominant method used for parentage analysis, single-nucleotide polymorphisms are becoming more prevalent (Flanagan and Jones 2019).

Several non-genetic methods can be used to link seeds to their parents. One approach is to use rapid real-time mass spectrum derived chemical fingerprints (Lesiak *et al*. 2015). Another is to label the flowers or seeds of a focal plant with N isotopes, then use mass spectrometry and a mixing model to determine what proportion of seeds collected in seed traps came from the focal plant (Carlo *et al*. 2009; Herrmann *et al*. 2016). Similarly, seeds at a source plant can be labelled with Gamma-emitting isotopes and re-located using a Geiger counter (Vander Wall 1992). More traditional approaches include marking individual seeds, for example using paint (Bullock *et al*. 2006).

#### Identifying the dispersal vector

In addition to knowing the parent plants and linking dispersed seeds to them, one must also know all dispersal vectors operating in the system. In simple systems, where a single vector is thought to operate, the TDK concept would encourage researchers to articulate that assumption and assess whether other dispersal processes might come into play (Higgins *et al*. 2003). For example, in wind-dispersed species, tumble dispersal (Zhu *et al*. 2019) or human-mediated dispersal (Wichmann *et al*. 2009) may also play a role. In more complex dispersal systems, identifying all dispersal vectors for a focal plant species poses a considerable challenge.

Linking seeds to their dispersal vectors for bird- or mammal-dispersed plants has typically required direct observation of trees and animal vectors or identification of scat using morphological characteristics, but both approaches have limitations. Direct observation is effective for diurnal frugivores that can be easily followed (e.g. primates, ants), but less effective for small-bodied, volant or nocturnal frugivores, or in areas that are difficult to traverse. Scat identification is useful for distinguishing between frugivore guilds (e.g. birds vs. mammals), but is less reliable for distinguishing between species within a guild. A recently developed empirical approach enables researchers to link seed source and vector identity using barcoding of defecated or regurgitated samples combined with genetic parentage analysis of the dispersed seeds. Studies have since used DNA barcoding in species discrimination of scat from *Canids* and other carnivores (Chaves *et al*. 2012; Rodríguez-Castro *et al*. 2018) as well as in frugivorous birds (González-Varo *et al*. 2014). This approach shows great promise for revealing the dispersal vectors comprising the TDK and in doing so, building a fruit–frugivore dispersal network. For the tree species, *Olea europaea* var. *sylvestris*, González-Varo *et al*. (2017) used parentage analysis and DNA barcoding of scat containing seeds to identify both the dispersers and seed origin.

Some commonly overlooked vectors include gravity dispersal of fleshy fruits that fall untouched by frugivores and movement by secondary and higher-order dispersers such as scatter-hoarding mammals and ants. Most studies of post-dispersal seed removal attribute the lost seeds to seed predation when many of those seeds might be secondarily dispersed and cached (see Gómez *et al*. 2019). Dispersal is often sequential; for example, many elaiosome-bearing species experience ballistic dispersal followed by ant dispersal (Vander Wall and Longland 2004), and agouti were found to have re-cached seeds up to 36 times (Jansen *et al*. 2012). Measuring secondary or higher-order seed dispersal, as well as identifying these dispersers, often requires a different set of methods than would be used for primary seed dispersal (Vander Wall *et al*. 2005; Gallegos *et al*. 2014). For example, Jansen *et al*. (2012) used miniature radio transmitters attached to seeds to track movement of seeds by scatter-hoarders. Knowledge of the pattern and order of sequential movements facilitates the characterization of the TDK, as described in the modelling section below.

One challenge to identifying all dispersal vectors is that vectors may vary temporally or spatially, so capturing the full suite of plant species a vector disperses or the full suite of vectors for a single plant species requires studies over multiple seasons, years and locations (Carnicer *et al*. 2009). To facilitate predictive understanding and generalizability across systems, it may be desirable to produce a set of nested TDKs reflecting different temporal resolutions (e.g. seasonal, annual, generational or lifetime TDKs) and different spatial resolutions (e.g. patch, landscape or across the entire species range) as well as different ecological levels (e.g. populations, species, functional groups).

#### Measuring dispersal across the landscape

Lagrangian approaches that build dispersal kernels by tracking seeds or animals carrying seeds have long been limited by the inability to track very small seeds or to follow animals across rugged or heterogeneous landscapes. However, tracking movement of animals or seeds across the landscape is getting easier through the development of better and smaller tracking devices and automated telemetry systems (Kays *et al*. 2015). GPS tracking is now possible for small to medium birds and mammals, and accelerometers paired with machine learning approaches allow identification of typical behaviours (e.g. feeding vs. moving) (Brown *et al*. 2013). While tracking is difficult for primary dispersal, it is even more challenging for secondary or higher-order dispersal (Nathan 2007). Seed dispersal by scatter-hoarding rodents can be tracked using very high frequency tags or passive integrated transponders tags placed on seeds (Hirsch *et al*. 2012a; Suselbeek *et al*. 2013), and the data on starting and ending points can then be used to fit dispersal kernels (Hirsch *et al*. 2012b). Another promising and innovative method used high-resolution imagery and automated image analysis to monitor seed dispersal by ants in a lab setting (Bologna *et al*. 2017). Epizoochorous seed dispersal is a challenge to study, because seed attachment and detachment is strongly affected by the animal’s behaviour, but lab studies using various animals and seed types have provided empirical data on attachment and detachment rates (Couvreur *et al*. 2004; Tackenberg *et al*. 2006; Will *et al*. 2007). Although technological advances have improved our ability to empirically measure dispersal, the field would benefit from coordinated efforts using standardized methods replicated across space and time.

Since establishing the TDK for a single species is time consuming, trying to estimate TDKs across a diverse plant and animal community is even more challenging. However, dispersal vectors, distances and the shape of the dispersal kernels can be inferred from traits of the dispersed species, based on observations, empirical studies and simulations. For example, if it is known that a plant species is dispersed initially by three bird species, then secondarily by ants, one could characterize TDK’s for the plant species using empirical movement data collected for plant and animal species with similar traits. Trait characteristics relating to dispersal have been collected and curated in regional [i.e. BROT 2.0 for the Mediterranean basin (Tavşanoğlu and Pausas 2018)), continental (i.e. LEDA Traitbase (Kleyer *et al*. 2008)] and global trait databases [i.e. TRY (Kattge *et al*. 2011) and SID (Royal Botanic Gardens Kew 2019)]. Dispersal-related traits in plants that are collected to varying completeness in these databases include: seed traits such as size, weight and terminal velocity, seed number, seed-release height and also phenological traits such as germination time. Databases have been successfully used to predict (maximum) dispersal distance (Tamme *et al*. 2014) and spread velocities for various species (Lustenhouwer *et al*. 2017), and have contributed to the understanding of LDD for plant distributions (Arjona *et al*. 2018). Traits can also be useful for classifying vectors and dispersing organisms into functional groups (Aslan *et al*. 2019). Assessing the relevant functional groups for which to develop TDKs requires extensive ecological and natural history data on which vectors are dispersing viable seeds and where those seeds are landing (Dennis and Westcott 2006). In recent years, there has been an increase in data collection necessary for identifying functional groups. In particular, an increasing number of well-characterized fruit–frugivore networks can identify functionally similar frugivores and fruits (Plein *et al*. 2013; Fontúrbel *et al*. 2015).

Environmental context such as wind speed and direction, geographic position, landscape composition and landscape configuration complicate the reconstruction of dispersal events (Shea 2007). While dispersal is often assumed to be isotropic and continuous, that assumption does not follow for many species; knowing how seeds move across a landscape mosaic is important for TDK characterization when dispersal is dependent upon the habitat. Dispersal is affected by landscape characteristics, which can create barriers or corridors, affecting seed movement directly or indirectly by influencing the movement and behaviour of vectors (Levey *et al*. 2005). Uncertainty about the route of dispersal events within the TDK can be addressed empirically by combining remote sensing, machine learning and genetics within a landscape ecology framework to identify dispersal networks without directly tracking seed movement (Balkenhol *et al*. 2019). Using parentage analysis and DNA barcoding of scat containing seeds collected in seed traps within continuous forest and matrix with isolated trees, González-Varo *et al*. (2017) identified unique spatial patterns of seed dispersal by each avian frugivore.

#### Measuring seed dispersal effectiveness

One of the biggest remaining challenges is to account for seed dispersal effectiveness (e.g. SDE; Schupp *et al*. 2010), which is measured by combining the *quantity* of seeds removed by each vector with the *quality* of dispersal based on the likelihood of establishment and growth following dispersal. Even when the natal source and vectors are known, researchers often face challenges closing the loop to link a dispersal event to the final outcome. Seed dispersal effectiveness can vary by vector or by the sequence of vectors; therefore, detailed seed fate studies are necessary to parse out the role of vectors (Rother *et al*. 2015). Molecular approaches can be used to assess the effective or realized seed dispersal by genotyping established seedlings and using parentage analysis to match seedlings to natal source (e.g. Moran and Clark 2012). Parentage analysis has successfully resolved dispersal distances in both abiotically (Piotti *et al*. 2009; Johnson *et al*. 2017; Monthe *et al*. 2017) and biotically (Hardesty *et al*. 2006; Ismail *et al*. 2017) vectored species, allowing dispersal kernels to be constructed. Including data on the quantity and quality of seed dispersal by each vector will make the TDK more useful for predicting changes in plant populations.

Overall, we propose that better estimations of dispersal will result from improved assessment of the contributions of multiple dispersal vectors to the TDK, advances in combining different methodological approaches, improved approaches to model rare LDD events, use of traits and trait databases to identify functional types, studies focusing on the role of heterogeneous landscapes in dispersal and an increased focus on SDE (Table 1).

### Mathematical or conceptual modelling of the TDK

The purpose of the TDK is to quantitatively describe the pattern of seed dispersal from source plant to the seed’s final resting point. A seed dispersal kernel (probability density function) is required for each vector–plant combination to describe the distribution of distances potentially travelled by an individual seed affected by that vector. Then these different processes must be combined correctly to describe the overall probability that an individual seed moves any given distance via an amalgam of processes (Jordano *et al*. 2007; Wichmann *et al*. 2009; Horvitz and Koop 2015). The TDK is useful as a single kernel representing all vectors when one wishes to predict dispersal in a given habitat with multiple dispersers present; however, the TDK is most useful when the individual vectors can be separated, as this allows exploration of how changes in one or multiple vectors may affect the overall TDK.

Overall, a TDK model needs to include information on how many seeds are produced, what proportion of those seeds are dispersed by each of the primary vectors, and where those seeds land, and then any secondary or higher-order dispersers would be modelled based on their effects on the subset of seeds that they disperse. The TDK can be modelled using a wide variety of analytical or simulation approaches spanning a range of complexity. The most appropriate method may depend on the quality and amount of data available.

In principle, the TDK may be constructed analytically using mathematical functions. Doing so is often feasible if the dispersal processes involved are easily sampled (e.g. seed trap data for a species dispersed solely by volant frugivores in a homogenous environment) and separately identifiable. Probability distributions commonly employed in the construction of dispersal kernels include the Gaussian, lognormal, inverse Gaussian and 2Dt (Clark *et al*. 1999; Jongejans *et al*. 2008; Nathan *et al*. 2012; Bullock *et al*. 2017). The density of seeds following a single simple dispersal event involving such a distribution can frequently be realized through the convolution of the seed density before dispersal and the probability distribution involved.

For most plant species, movement takes place both in parallel (e.g. dispersal of seeds from a parent plant by both wind and an avian disperser, which may occur independently) and in series involving secondary and higher-order dispersal (e.g. dispersal of seed by wind, followed by dispersal of the same seed by an ant), where the later movements occur following primary dispersal (Fig. 1a). When processes are parallel, it is straightforward to express the joint kernel as a weighted mix of the individual seed dispersal kernels (Higgins *et al*. 2003). For a sequential process, the overall kernel is derived by the mathematical composition of the different dispersal kernels for each of the dispersal steps. Frequently, these compositions are convolutions of intermediate seed densities and probability distributions. Once this is done for each sequential process, the resulting composite processes can be considered in parallel and their kernels added with appropriate weights according to the proportion of seeds dispersed through each combination of dispersal modes (Neubert and Parker 2004). The result is the TDK. Gravity models for dispersal, which explicitly consider source, relocation and destination processes, may be extended to include multiple vectors (Jongejans *et al*. 2015). Simulation approaches can be used to estimate TDKs (and TEDKs) in the framework of a cellular automaton or individual-based models.

The characterization of dispersal kernels may be affected by landscape heterogeneity, which is a key challenge for modelling. More complex dispersal kernels are required in heterogeneous habitats (Neupane and Powell 2015). The WALD or WINDISPER models (Nathan *et al*. 2011) are useful for mechanistically modelling wind dispersal, but cannot solve all inherent fluid dynamics issues in a non-trivial, heterogeneous environment. For example, there is reason to believe (i) that wind drift is not always linear, (ii) that drift is not always constant in time and (iii) that drift varies spatially—not just in directions parallel to the ground, but also with altitude (Stephenson *et al*. 2007; Nathan *et al*. 2011). However, some mechanistic models have been developed that can incorporate the effect of landscape on seed dispersal patterns (Soons *et al*. 2004). Trakhtenbrot *et al*. (2014) show that dispersal distance and direction estimates are sensitive to the terrain in a mechanistic wind dispersal model and Shea *et al*. (2008) describe movement in water currents. The Stochastic Movement Simulator can be used for modelling animal movement in a heterogeneous environment (Palmer *et al*. 2011) and account for interactive effects of landscape and species’ dispersal behaviour (Bocedi *et al*. 2014).

### Statistical challenges to estimating or parameterizing the TDK

As described above, the TDK emerges as a mix of the contributions of the individual dispersal vectors. Existing statistical approaches allow parameterizing individual dispersal kernels from multiple, separate data streams (e.g. tracking seeds per vectors, trapping seeds), as it is already commonly done in integrated population models (Schaub and Abadi 2010). In the TDK framework, the kernels of all dispersal vectors (the sub-kernels) need to be combined to form the TDK (Fig. 1c). When a single sampling method (e.g. seed rain traps) is used to capture the TDK, the sub-kernels of each dispersal vector must be disentangled. In principle, it is straightforward to reflect both cases in a statistical model. However, estimating these statistical models and correctly assigning the contribution of each dispersal vector to the TDK is not trivial. Here, we highlight five statistical issues that we view as crucial for successfully applying the total dispersal concept to real data.

The first issue is that separating the contributions of the various dispersal vectors from the observed total kernel alone is very difficult, whereas it is less of a problem to construct a TDK from known contributions of different vectors. It is thus necessary to obtain good prior information on the kernels of the individual dispersal vectors, either from general ecological knowledge [e.g. that wind dispersal usually has a much wider kernel than ballistic dispersal (Bullock *et al*. 2017)], or from targeted studies for each of the vectors as described above. Empirical approaches often fail to capture rare LDD events, which leads to poor characterization of the tail of the distribution. However, a promising new approach applies ‘statistics of extremes’ to estimate these disproportionately important LDD events (García and Borda-de-Água 2016). In addition, dispersal from outside the mapped area can be estimated using statistical models (Oddou-Muratorio *et al*. 2010; e.g. Moran and Clark 2011). Given adequate data to characterize the TDK, Bayesian statistical methods would allow the mixing of prior information on the individual dispersal vectors with observations of the total kernel. The virtual ecologist approach uses simulated data to qualify analysis tools and sampling methods (Zurell *et al*. 2010), and could be used to identify which vectors require improved empirical data (e.g. larger samples sizes, or higher sampling frequency) for quantification.

**Figure 2.**
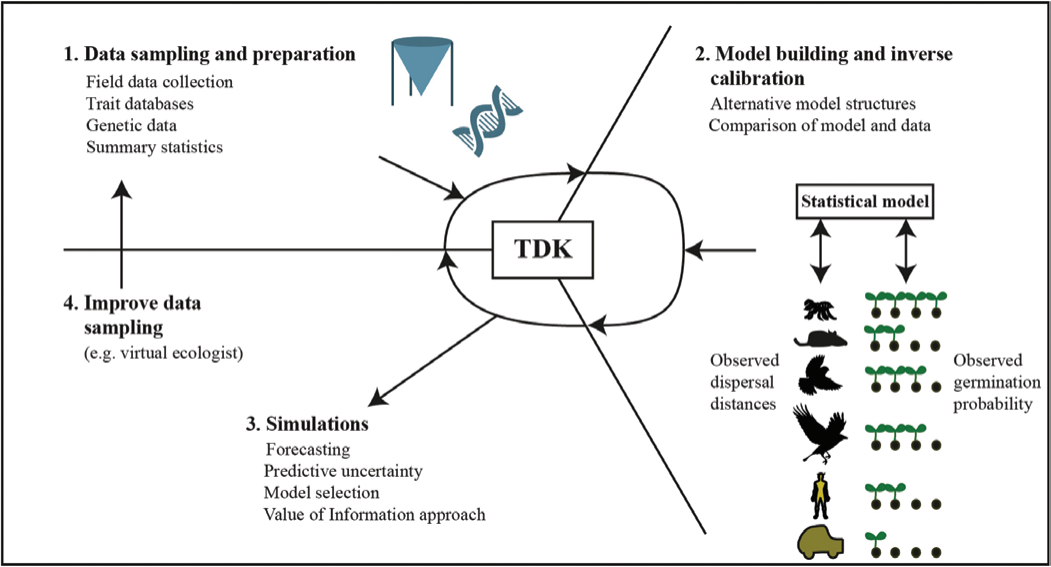
Schematic representation of the modelling cycle for parameterizing and selecting total dispersal kernel models. It includes (i) data sampling and preparation, (ii) model building and (iii) simulations, for example projections of spread (Neubert and Caswell 2000) or different management scenarios (Shea *et al*. 2014). Simulations can be used for improving the sampling design (iv) using a virtual ecologist approach (Zurell *et al*. 2010), thereby restarting the cycle.

A second problem is how to deal with parameter estimation in high-diversity systems, where there are a large number of biotic and abiotic dispersal vectors. Similar problems arise in many areas of ecology, and typical solutions are to either (i) group plant or animal species *a priori* into functional groups according to their traits or phylogenies (Aslan *et al*. 2019), or (ii) fit hierarchical statistical models, where species that are close according to their phylogenies or traits are assumed to have similar properties (Mokany *et al*. 2014). These solutions should work equally well (with the known limitations) for the problem of characterizing total seed dispersal kernels. We recommend to first fit the dispersal kernels of the biotic and abiotic vectors independently, if possible. If it is only possible to group or jointly estimate dispersal kernels as described, it can be useful to test the model or simulation outputs against data to validate the model structure. Such tests can be done via formal methods for simulation-based inference (e.g. Hartig *et al*. 2011), or via informal comparisons of multiple empirical patterns against model outputs (Grimm and Railsback 2005, 2012). For example, dispersal distances observed for seeds of endozoochorous plants result from the movement capacity of the dispersal vectors and observed germination rates resulting from gut passage; when used in tandem, these patterns provide more information on underlying processes than any one pattern alone and can thus help to select appropriate model structure and parameters (Fig. 2).

A third problem is to account for sequential dispersal processes, either via several dispersal steps of the same vector (e.g. wind), or through a combination of primary, secondary, and higher-order dispersal vectors. Similar to the first issue, it will be advantageous to estimate each sub-process independently, and then combine those direct estimates with observations of the total kernel, if such observations can be obtained. For the latter joint hierarchical model, there is no suitable off-the-shelf software, but hierarchical models of this type can be estimated using a Bayesian framework (e.g. Bastida *et al*. 2018). As described above, such model fitting needs to represent the convolution of dispersal kernels and not a simple multiplication.

Fourth, certain vectors can create clustered dispersal patterns, such as in faeces or beneath sleeping trees (Schupp *et al*. 2002), and statistical models thus have to account for spatial correlations between seeds. Some advanced statistical software packages, such as INLA (Rue *et al*. 2009; Martins *et al*. 2013) allow the fitting of spatial point processes, although it should be noted that the capability of off-the-shelf software for this problem is limited, in particular when combined with the third issue (hierarchical dispersal processes).

Finally, incorporating seed fate is necessary for understanding SDE and thus the TEDK, and the TDK is a great framework for linking effectiveness to dispersal vectors. However, considering post-dispersal seed fate increases the complexity enormously, as physiological and demographic processes unrelated to dispersal also need to be taken into account. As mentioned above, one could infer survival probabilities for seeds dispersed different distances or into different environments by comparing the distribution of seeds to that of seedlings using inverse modelling or genetic approaches. Seedling emergence probabilities can also be estimated using population means as in traditional matrix population models or full probabilities as in integral projection models, which account for uncertainty around the emergence parameter (Easterling *et al*. 2000) and have spatial analogues that explicitly incorporate dispersal kernels (Jongejans *et al*. 2011). Species distribution modelling (SDM) literature has discussed combining SDMs, demographic models, and dispersal (Ehrlén and Morris 2015); while the aim is slightly different, some of these models could be adapted to the TEDK framework. Hierarchical Bayesian modelling is well equipped to deal with combining prior probabilities of different dispersal vectors plus germination, and spatial point processes.

#### Using the TDK framework to address critical questions in dispersal ecology

The TDK is the first step towards understanding the role of dispersal or the impact of changes in dispersal on plant populations and communities. We can assess the importance of different dispersal pathways to the TDK using model selection, either based on statistical methods, or on Value of Information methods (Runge *et al*. 2011). Model versions including and excluding particular vectors or dispersal pathways can be compared. For example, if a model that excludes a particular vector predicts the same TDK as a model variant that includes that vector, then the vector is relatively unimportant in that system. Conversely, a disperser whose exclusion generates major changes in the TDK is clearly a critical component of the dispersal network for the focal species. This is akin to network approaches that explore the impact of simulated extinctions on the rest of the network (Schleuning *et al*. 2016); with increased adoption of the TDK approach, we may eventually be able to use similar meta-analytic approaches to identify factors conferring robustness to particular species or functional groups.

Simulations based on alternative model structures could also be used to address the conservation implications of lost vectors or shifts in the relative abundance among vectors in the face of habitat loss, species declines or invasions or climate change (Mokany *et al*. 2014). More nuanced analyses could be conducted if there are expectations that a vector’s dispersal kernel might change, for example in future climates—sensitivity analyses to anticipated changes could be conducted by alteration of the functional form or parameterization of the dispersal kernel (Bullock *et al*. 2012). This would also allow us to address the potential for currently unimportant pathways or dispersal vectors to become important under changing environmental conditions.

Similar methods could be used to assess the potential impact of uncertainty about kernels either in the present (due to missing or incomplete data) or in the future (in response to different environmental conditions or community changes) on management recommendations for endangered, harvested or invasive species. For example, Shea *et al*. (2014) provide a template that can be extended to the TDK in their study on the optimal control of a disease outbreak with uncertainty about the (single) dispersal kernel. If uncertainty about some dispersal pathways makes a bigger impact on management recommendations than others, then the value of learning about the former is highlighted (formally this is a Value of Information analysis, analogous to a sensitivity analysis for management; Runge *et al*. 2011). Such approaches could also be used to examine the potential impact of new dispersal pathways (e.g. assisted migration or new dispersers). For example, it has recently been found that plastic detritus in the ocean serves as a stable substrate for invasive species to spread following the recent Japanese tsunami (Carlton *et al*. 2017)—models including increases in such contamination could assess the inherent risk.

Ultimately, we would not only like to understand where seeds land as described by the TDK, but also how the TDK, and thereby the role of dispersal, affects population and community dynamics and species coexistence. Well-developed mathematical biology models link dispersal to population dynamics by coupling dispersal with reproduction in reaction-diffusion models (Fisher 1937; Skellam 1951; Okubo and Levin 2001). Such models assume geometric growth and can be realized in the context of integrodifferential models with exponential dispersal kernels, although other kernels can be used. Using an integrodifference and/or integrodifferential equation model framework (Neubert *et al*. 1995), we can construct a total effective dispersal kernel that additively incorporates all distinct seed transmission pathways, where each individual pathway is a series composition of vector-mediated movements. Another commonly used approach for modelling population dynamics is that of metapopulation models, which implicitly include a total effective dispersal kernel, and enable exploration of the role of suitable and unsuitable habitat in patches across a landscape. One must be wary of equating the TDK with the TEDK, because one of the biggest remaining empirical challenges in seed dispersal ecology is linking seed dispersal to plant fitness by tracking seed and seedling fate. We hypothesize that the TDK rarely resembles the total effective dispersal kernel due to distance-dependent mortality, heterogeneous habitat and microhabitat quality, and the effect of gut passage on germination. Because seed fate is often linked to the dispersal vector, it is important to understand the effectiveness of each vector rather than simply measuring the TDK without identifying how seeds reached their final destination.

#### Combining empirical, statistical and mathematical approaches

Estimating the TDK and the TEDK effectively requires an adaptive process that combines empirical, conceptual modelling, and statistical approaches. The various components inform the outcome and can and should be adapted to respond to uncertainty, information gaps and temporal or spatial changes in conditions. In our discussion, we have so far followed a classical sequence of data collection, models that allow predictions and statistical analysis. However, these steps will often be reversed or combined. For example, models can be a final step in the cycle or a new starting point for directing the field sampling, especially when researchers face logistical or financial constraints (Shea *et al*. 2008), as information from TDK models can guide future observational efforts or inform the design of targeted experiments (Fig. 2). When prioritizing research based on preliminary data, models can be used to simulate the field sampling, and identify which vectors really matter and thus need to be measured thoroughly (*sensu* the ‘virtual ecologist’ approach (Zurell *et al*. 2010)). A particular example is the work of Skarpaas *et al*. (2005), which used models to test common alternative trap layouts for estimating dispersal kernels of a wind-dispersed plant, finding that some common trap designs were essentially useless. The model results were then used to design the trap layout to successfully measure dispersal in the field (Skarpaas and Shea 2007). Analogously, if model selection identifies two or more TDK models as similarly important in terms of explanatory power, then a virtual ecologist approach could be used to identify data (e.g. distance classes) that would aid selection of alternative model structures. Thus, models can accelerate knowledge gain about important processes, closing the modelling cycle (Fig. 2; Jeltsch *et al*. 2013).

## Conclusions and Outlook

The importance of being able to calculate the TDK has been recognized and acknowledged by the dispersal ecology community (Nathan *et al*. 2012; Beckman and Rogers 2013). Inclusion of multiple dispersal vectors and their respective contributions to SDE improves TDK characterization and predictions about how plant populations will respond to global change drivers. There are still many challenges, chief among them the recognition that the landscape often influences dispersal, LDD is a challenge to accurately quantify, and that an understanding of seed fate is required to link dispersal to population dynamics and spread. However, computational, experimental and empirical techniques are constantly improving and becoming less expensive, with technological advances and increased data availability to estimate outcomes of seed dispersal (Table 1). We thus believe that quantifying the TDK and ultimately, the TEDK, will become feasible through the combination of information from different data sources.

**Table 1.** Empirical challenges associated with fitting total dispersal kernels, novel approaches to resolve the challenges and case studies demonstrating how this approach has been used to fit dispersal kernels or highlighting technology that could be used to study seed dispersal.

Challenge	Approaches	Example
Locating all possible parent plants	Remote sensing	Baldeck *et al.* (2015), Andrew and Ustin (2010)
Linking dispersed seeds to parent plants	Genetic parentage analysis of seeds, chemical fingerprints, nitrogen or gamma isotopes	Jordano *et al.* (2007), Lesiak *et al.* (2015), Herrmann *et al.* (2016), Vander Wall (1992)
Locating all possible dispersal vectors	Frugivory observations, faecal samples	Jansen *et al.* (2014)
Linking dispersed seeds to dispersal vector	Genetic analysis of scat	Gonzalez-Varo *et al.* (2014)
Capturing movement of dispersal vectors	Improved transmitters	Wikelski (2010)
Estimating TDKs across a community	Functional traits	Mokany *et al.* (2014)
Linking movement to dispersal effectiveness	Genetic parentage analysis of seedlings	Moran and Clark (2011)

Finding a common language might support these endeavours. As a start, we propose the adoption of the phrase ‘total dispersal kernel’ instead of ‘complete’ or ‘full’ dispersal kernel, primarily because this term is already the most widely used among the three. Calls for generalizations (e.g. Jongejans *et al*. 2015) remain largely unanswered, and new generalizations or approaches to better predict dispersal have been introduced (e.g. Sadlo *et al*. 2018), without employing a common language. We hope this paper serves to reinvigorate the study of TDKs through improved integration of empirical, mathematical, and statistical approaches. Identifying the factors that that determine where seeds land has been a longstanding challenge for plant and community ecology; advances in characterizing the TDK are broadly relevant for ecology and also to conservation practitioners trying to manage for ecological resilience in a changing world.

## Sources of Funding

Our discussion emerged from a National Science Foundation-funded Seed Dispersal Workshop (DEB 1548194) held at the National Socio-Environmental Synthesis Center in May 2016. D.Z. received funding from the Swiss National Science Foundation (SNF, grant: PZ00P3_168136/1) and from the German Science Foundation (DFG, grant: ZU 361/1-1). J.B. received funding from CEH National Capability NEC06895.

## Contributions by the Authors

H.R. led the writing of the manuscript. All authors contributed to concept development and writing and are listed in alphabetical order within each level of contribution.
